# Design of Quad-Port MIMO/Diversity Antenna with Triple-Band Elimination Characteristics for Super-Wideband Applications

**DOI:** 10.3390/s20030624

**Published:** 2020-01-22

**Authors:** Pawan Kumar, Shabana Urooj, Fadwa Alrowais

**Affiliations:** 1 Department of Electrical Engineering, School of Engineering, Gautam Buddha University, Greater Noida 201308, India; pawangupta.iitb@gmail.com; 2 Department of Electrical Engineering, College of Engineering, Princess Nourah Bint Abdulrahman University, Riyadh 84428, Saudi Arabia; 3 Department of Computer Sciences, College of Computer and Information Sciences, Princess Nourah Bint Abdulrahman University, Riyadh 84428, Saudi Arabia; fmalrowais@pnu.edu.sa

**Keywords:** diversity, isolation, MIMO, monopole, SWB

## Abstract

A compact, low-profile, coplanar waveguide (CPW)-fed quad-port multiple-input–multiple-output (MIMO)/diversity antenna with triple band-notched (Wi-MAX, WLAN, and X-band) characteristics is proposed for super-wideband (SWB) applications. The proposed design contains four similar truncated–semi-elliptical–self-complementary (TSESC) radiating patches, which are excited through tapered CPW feed lines. A complementary slot matching the radiating patch is introduced in the ground plane of the truncated semi-elliptical antenna element to obtain SWB. The designed MIMO/diversity antenna displays a bandwidth ratio of 31:1 and impedance bandwidth (|S_11_| ≤ − 10 dB) of 1.3–40 GHz. In addition, a complementary split-ring resonator (CSRR) is implanted in the resonating patch to eliminate WLAN (5.5 GHz) and X-band (8.5 GHz) signals from SWB. Further, an L-shaped slit is used to remove Wi-MAX (3.5 GHz) band interferences. The MIMO antenna prototype is fabricated, and a good agreement is achieved between the simulated and experimental outcomes.

## 1. Introduction

In contemporary wireless communication, the demand for super-wideband (SWB) and ultra-wideband (UWB) antennas is on the rise [1,2]. The UWB antenna possesses a bandwidth ratio of 3.4:1, and its bandwidth is defined from 3.1 to 10.6 GHz (by the Federal Communications Commission) [3], while the SWB antenna offers a bandwidth ratio of more than 10:1 [4,5]. As compared to the UWB systems, the SWB antenna can be used for both short-range and long-range communication. The planar monopole antenna, owing to its small size, light weight, low cost, and ease of fabrication and integration, is a suitable candidate for obtaining UWB/SWB [6,7]. In the literature, several antennas with fractal geometry have been proposed for SWB applications. In [8], a coplanar waveguide (CPW)-fed hexagonal-shaped patch antenna modified by Sierpinski square fractal form was designed for a bandwidth ratio of 11:1. In [9], an antenna comprising modified star-triangular fractal (MSTF) geometry fed by a microstrip line and a semi-elliptical ground surface was reported. A CPW-fed octagonal-shaped radiating patch modified using four fractal iterations was proposed in [10]. In [11], an octagonal-shaped radiating patch antenna using the second iteration of the fractal shape was suggested. A monopole antenna comprising an egg-shaped radiating patch and a ground plane loaded with complementary semi-elliptical shaped fractal slot was developed in [12]. However, SWB antenna configurations using fractal shapes are difficult to manufacture, and practically only a few iterations are possible to design.

Recently, the use of self-complementary antenna (SCA) structures has been in focus for SWB and UWB communication systems. In [13], the authors proposed a semi-circular shaped quasi self-complementary (QSC) monopole antenna for UWB. In [14], a CPW-fed antenna composed of QSC geometry and a tapered radiating slot was designed for UWB. A microstrip line-fed monopole antenna comprising a quarter-circular disc and ground plane embedded with a quarter-circular slot was suggested for UWB systems [15]. In [16], an antenna with two parallelly arranged circular elements was presented with multiple-input–multiple-output (MIMO) characteristics. In [17], a UWB MIMO antenna containing two QSC radiating patches located opposite to each other to realize high isolation was proposed. In [18], a dual-port MIMO antenna with a castor leaf-shaped structure and possessing WLAN and Wi-MAX band rejection characteristics was reported. In [19], the authors presented a UWB MIMO antenna with two QSC half-circular monopoles, where the notch band and isolation were obtained by introducing parasitic strips of Levy’s and Hilbert fractal-shaped strips, respectively. A dual-port SWB MIMO antenna composed of two circular patches, asymmetrical E-shaped stubs, and mushroom-shaped electromagnetic band-gap (EBG) structures was suggested in [20]. In [21], a four-port SWB MIMO antenna with QSC resonating elements and exhibiting WLAN and Wi-MAX bands elimination characteristics was proposed. However, designs of the SWB antenna reported up until now primarily consist of antennas with one radiating element possessing one or two bands rejection characteristics. SWB antennas with four radiating elements and triple or multiple band elimination characteristics have seldom been reported.

In this article, a quad-port MIMO/diversity antenna consisting of four similar truncated–semi-elliptical–self-complementary (TSESC) radiating elements is presented. The resonating elements are excited through tapered CPW feed lines. A complementary slot matching the radiating patch is introduced in the ground plane of the truncated semi-elliptical antenna element to obtain SWB. The proposed resonating element displays a large bandwidth, which could be helpful for achieving a high data transmission rate, and the MIMO/diversity system offers better signal reception. The SWB antenna is designed to achieve triple elimination characteristics to avoid interferences of Wi-MAX, WLAN, and X-band signals. The Wi-MAX band interferences are rejected by introducing an L-shaped slit in the resonating patch. Similarly, a complementary split-ring resonator (CSRR) is introduced in the radiating element of the antenna for eliminating WLAN and X-band signals. The adjacent resonating elements are arranged orthogonally to each other, and diagonal elements are positioned in an anti-parallel manner to reduce coupling between the four radiators. The ground surfaces of the four monopole antenna unit cells are connected to ensure the same voltage in the proposed MIMO/diversity antenna.

## 2. Antenna Design

The input impedance of an SCA is constant, as shown by Mushiake’s relationship [22]:

(1) $$Z_{in}=\;\frac{Z_{0}}{2}\;\approx 188.5\;\Omega$$

where *Z*_0_ is the value of impedance measured in free space. The equation shows that the antenna dimensions, bandwidth, or wavelength do not affect the input impedance of a well-matched SCA. This method is used to design antennas with large bandwidth requirements [23,24].

### 2.1. TSESC SWB Antenna

**Figure 1:**
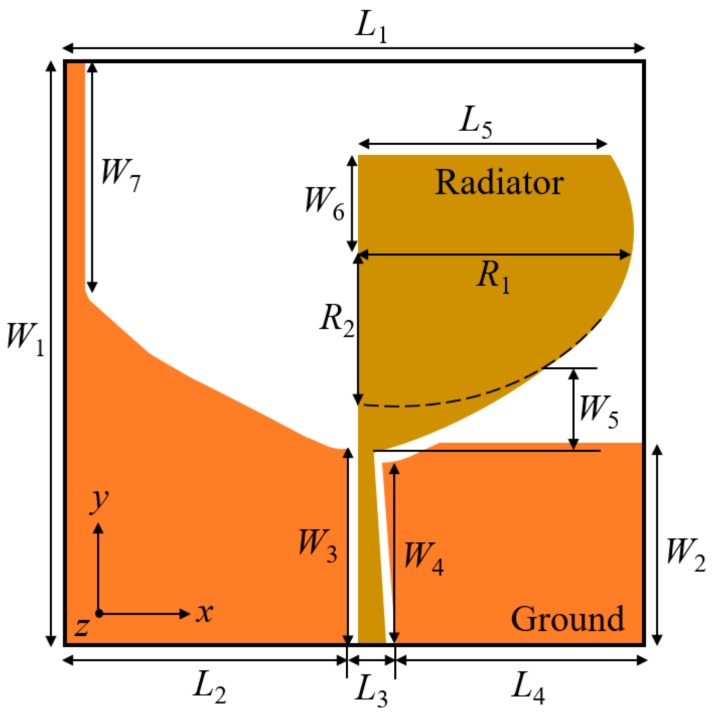
Layout of the truncated–semi-elliptical–self-complementary (TSESC) resonating element.

The schematic of the TSESC resonating element is illustrated in Figure 1. The design contains a truncated semi-elliptical monopole antenna excited by a tapered CPW feedline. A truncated semi-elliptical slot (corresponding to the radiating patch) is embedded in the ground plane of the antenna element to obtain SWB. The antenna is printed on the FR-4 dielectric substrate with a relative permittivity (*ε_r_*) 4.4, loss tangent (tan *δ*) 0.02 and 1.6 mm thickness. The TSESC resonating antenna element dimension details are presented in Table 1. The designing and optimization of the TSESC antenna are carried out using the ANSYS HFSS^®^ tool.

**Figure 2:**
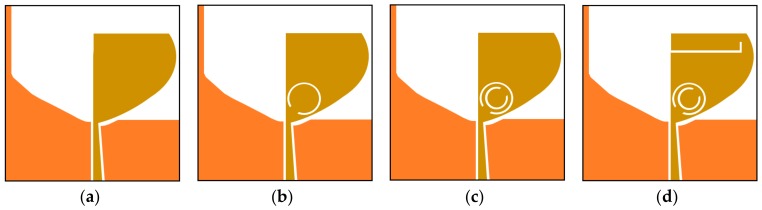
Geometrical design stages of the resonator: (**a**) stage i; (**b**) stage ii; (**c**) stage iii; (**d**) stage iv.

**Figure 3:**
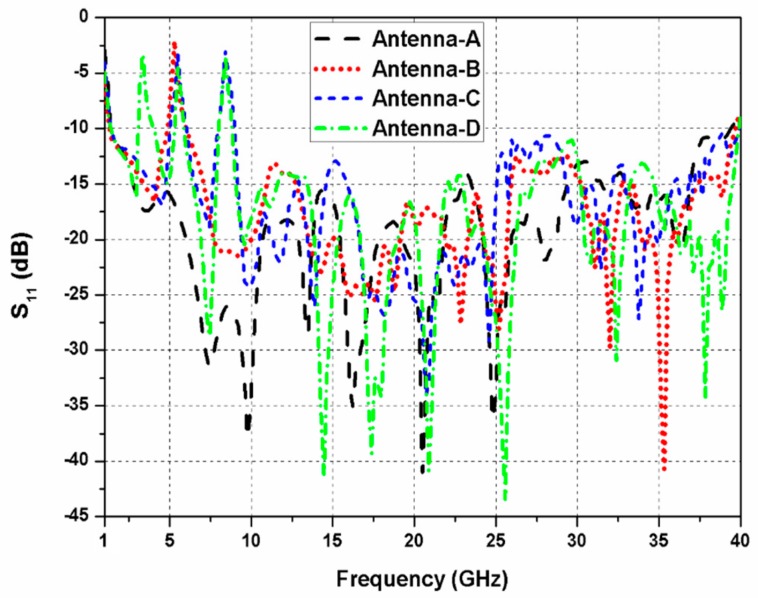
Reflection coefficients of the geometrical design stages.

The design stages of the resonating element are revealed in Figure 2. Initially, a truncated semi-elliptical shaped monopole radiator with a modified ground surface (Antenna-A) is designed, as displayed in Figure 2a. A radiating patch matching slot is etched from the ground surface of the antenna element to attain impedance matching over the SWB. The reflection coefficients of the geometrical design stages are presented in Figure 3. The designed antenna displays an impedance bandwidth (|S_11_| ≤ − 10 dB) of 1.3–40 GHz. In Figure 2b, a split-ring resonator (SRR) is laden on the resonating patch of the antenna element (Antenna-B) to eliminate interfering WLAN band (5.5 GHz) from the SWB. Next, as illustrated in Figure 2c, another SRR (complementary to the SRR in stage-ii) is laden on the radiating patch element (Antenna-C) to notch interfering X-band signals (8.5 GHz). Further, as demonstrated in Figure 2d, an L-shaped slit is introduced in the resonating patch (Antenna-D) to eliminate Wi-MAX band (3.5 GHz) interferences from SWB.

**Figure 4:**
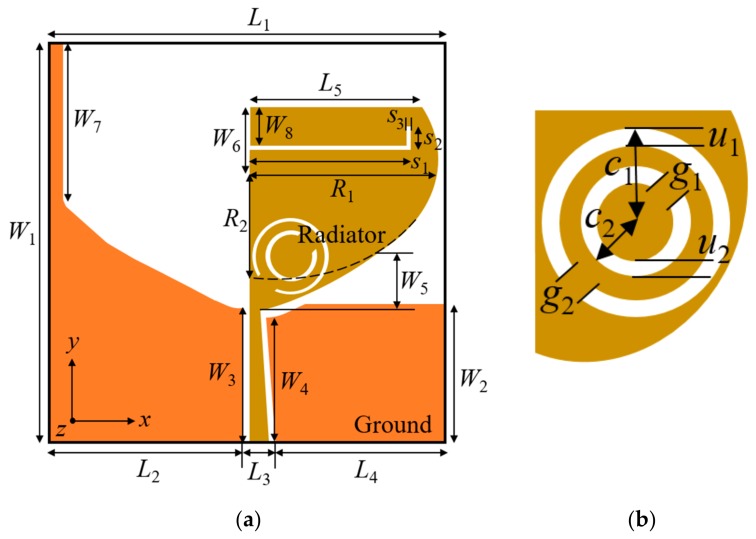
Proposed TSESC resonating element: (**a**) geometric layout; (**b**) magnified view of the implanted complementary split-ring resonator (CSRR).

The geometric layout of the proposed TSESC resonating element with the L-shaped slit and CSRR is shown in Figure 4a. The etched CSRR (for eliminating WLAN and X-band signals) is composed of two concentric circular rings of different radii and the same width, as shown in Figure 4b. The effective lengths of L-shaped slit (*S_L_*) and SRR (*S_Ri_*) are 0.29*λ_g_*_1_ and 0.52*λ_gi_*, respectively, which are calculated as [25]:

(2) $$S_{L}=\left(s_{1}+s_{2}+s_{3}\right)\approx 0.29\lambda_{g1}$$

(3) $$S_{R1}=\left\{\pi \left(2c_{1}-u_{1}\right)-g_{2}\right\}\approx 0.52\lambda_{g2}$$

(4) $$S_{R2}=\left\{\pi \left(2c_{2}-u_{2}\right)-g_{1}\right\}\approx 0.52\lambda_{g3}$$

(5) $$\lambda_{gi}=\frac{c}{f_{ci}}\left(\frac{1}{\sqrt{\varepsilon_{r,eff}}}\right);\;i=1,\;2,\;3$$

(6) $$\varepsilon_{r,eff}=\frac{\varepsilon_{r}+1}{2}$$

where *ε_r_* is the dielectric constant, *ε_r,eff_* is the effective dielectric constant, *c* is the velocity of light in free space, *f_ci_* is the centre frequency, and *λ_gi_* is the guided wavelength of the notched band.

**Figure 5:**
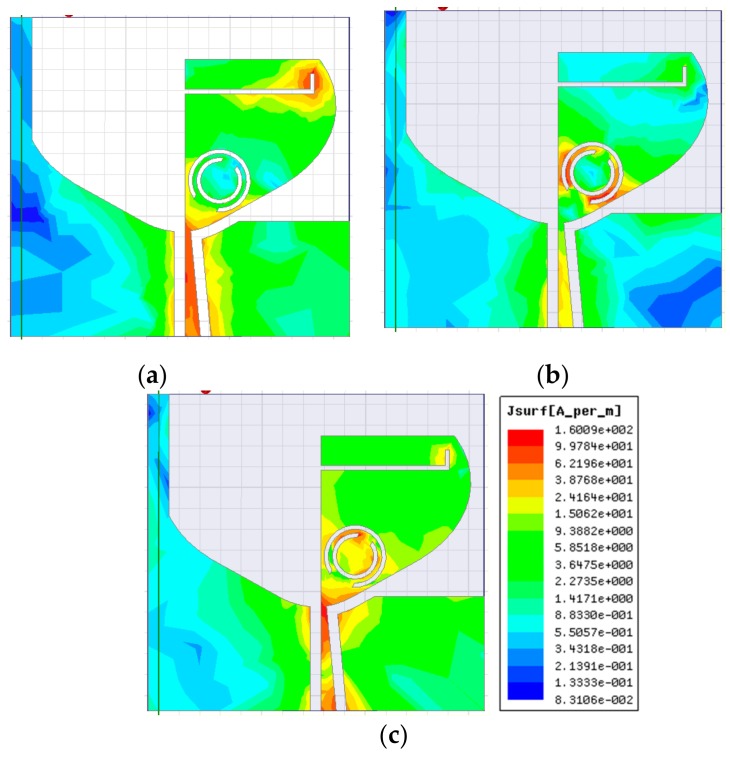
Surface current distribution of the antenna at (**a**) 3.5 GHz; (**b**) 5.5 GHz; and (**c**) 8.5 GHz.

Figure 5a–c signifies the surface current distributions at frequencies 3.5, 5.5, and 8.5 GHz, respectively. It is revealed in Figure 5a that the current is mainly concentrated along the L-shaped slit, which is accountable for the elimination of Wi-MAX band. In Figure 5b, a stronger current is seen near the outer split-ring, which results in the rejection of WLAN band. In the same way, the current is stronger close to the inner split-ring (illustrated in Figure 5c), which is accountable for the elimination of X-band signals. Therefore, by etching the L-shaped slit and CSRR from the TSESC antenna element, triple band-notched characteristics are obtained in the SWB.

### 2.2. TSESC SWB MIMO Antenna

**Figure 6:**
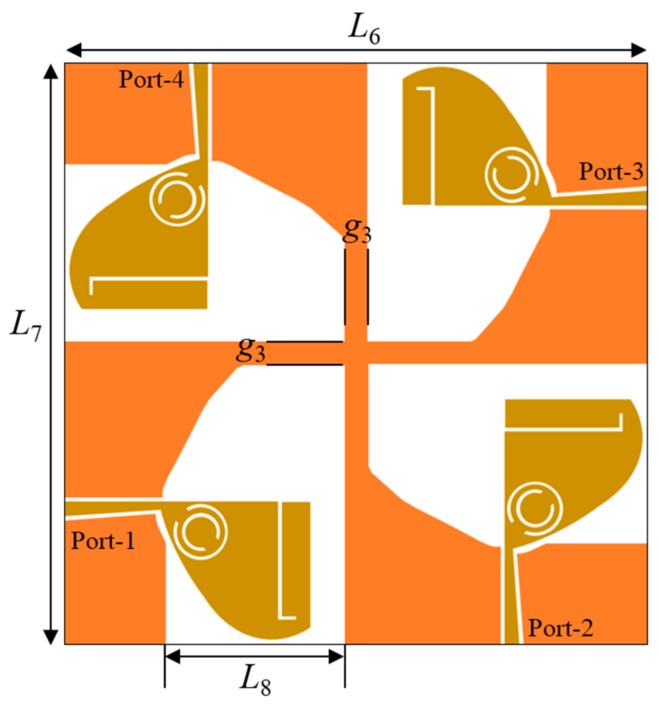
Geometric layout of the proposed TSESC multiple-input–multiple-output (MIMO) antenna.

**Figure 7:**
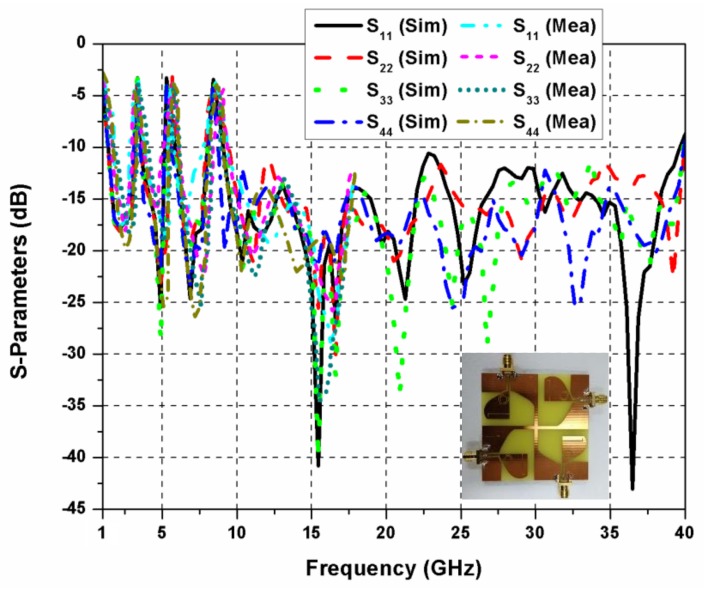
Reflection coefficients of the proposed quad-port super-wideband (SWB) MIMO antenna.

The antenna dimensions must be as small as possible due to space constraints in the communication devices. Designing the four-port diversity antenna is complex due to the mutual coupling of each radiating element to the other three similar resonating structures. The existence of multiple identical elements in the MIMO antenna leads to a manifold increase in the envelope correlation coefficient (ECC) and mutual interference among different elements. A four-port TSESC MIMO antenna with compact size is proposed for SWB applications. The geometric layout of the proposed antenna is presented in Figure 6, and the dimensions of various design parameters are provided in Table 1. The four elements of the quad-port antenna are arranged orthogonally to each other and the diagonal elements are positioned in an anti-parallel manner. The ground surfaces of the four monopole resonating elements are connected to induce the same voltage in the ground plane of the proposed MIMO/diversity antenna. The fabricated prototype of the MIMO antenna is demonstrated in Figure 7. The size of the proposed SWB MIMO/diversity antenna is 63×63×1.6 mm^3^.

## 3. Results 

**Figure 8:**
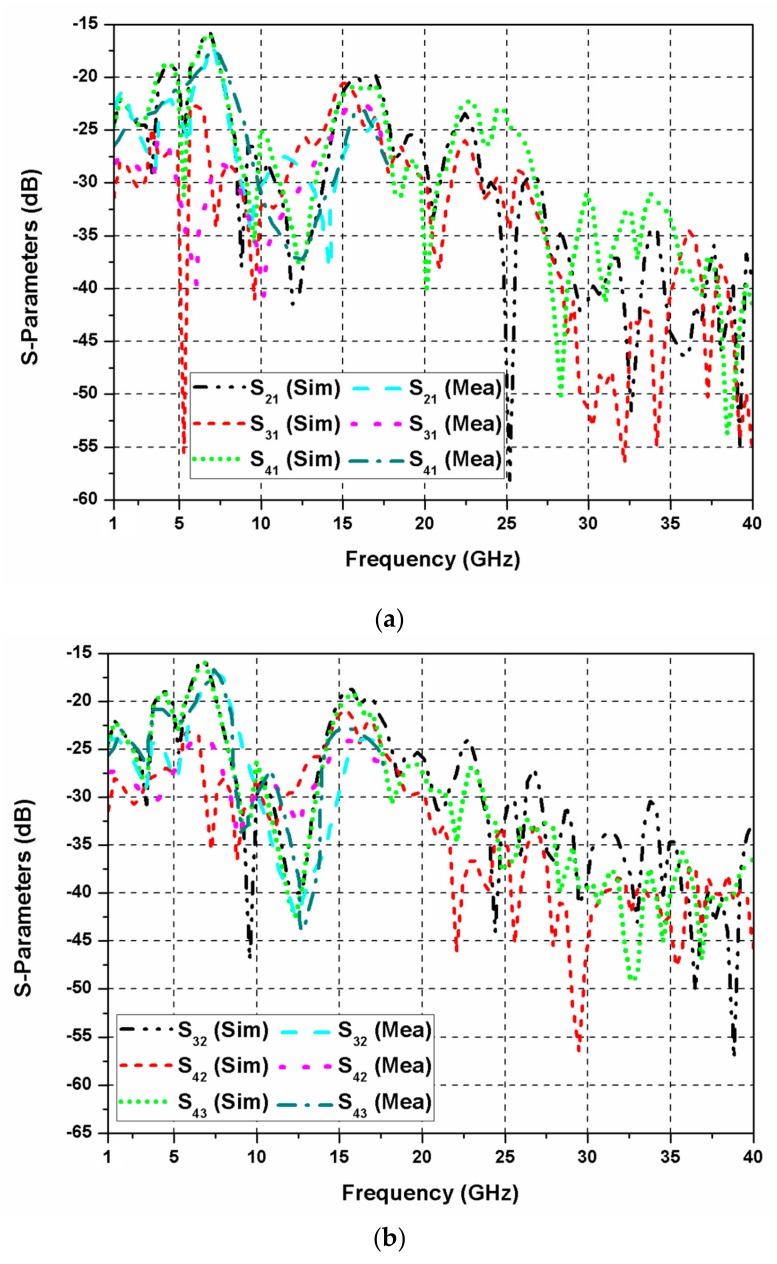
S-parameters of the TSESC MIMO antenna at (**a**) port-1; (**b**) other port.

**Figure 9:**
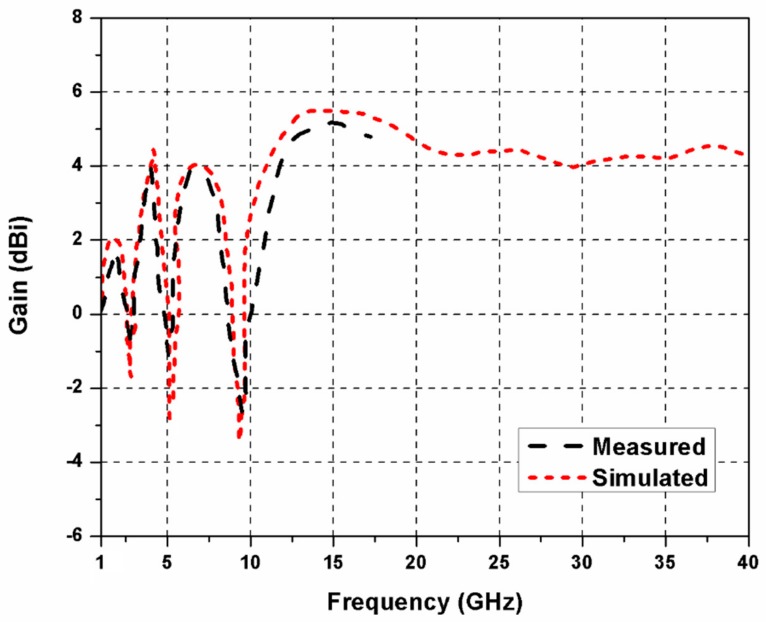
Simulated and measured gain of the SWB MIMO antenna.

The reflection coefficients of the proposed SWB MIMO antenna are shown in Figure 7. The impedance bandwidth and bandwidth ratio of the SWB MIMO antenna are 1.3–40 GHz and 31:1, respectively. The rejection of frequencies 3.5, 5.5, and 8.5 GHz is observed due to the introduction of L-shaped slit and CSRR in the radiating element of the antenna. In the proposed SWB antenna, the rejection bands can be controlled by changing sizes of the L-shaped slit and CSRR. The experimental results are shown only up to 18 GHz, which is due to the availability of ordinary SMA connectors and limited resources. While measurements are done at one port of the diversity antenna, the other ports are terminated using 50 Ω matched loads. Figure 8a,b demonstrates mutual coupling among different antenna elements of the proposed quad-port MIMO antenna. Isolation greater than 16 dB is obtained at lower frequencies, which increases significantly on shifting to higher frequencies. Figure 9 demonstrates that a peak gain of 5.5 dBi is realized. The antenna gain shows a sharp dip at the triple-band rejection frequencies; otherwise, it exhibits satisfactory behavior at other frequencies.

**Figure 10:**
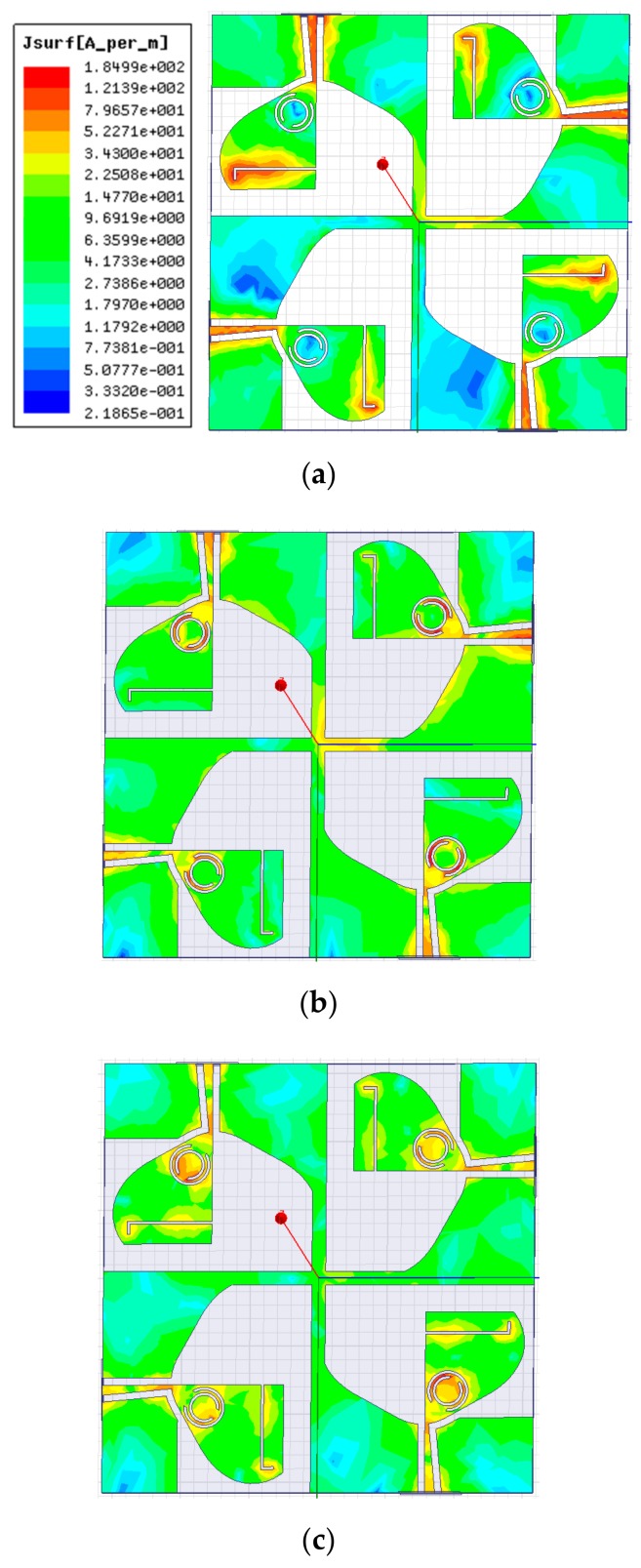
Simulated surface current distributions of the MIMO antenna at (**a**) 3.5; (**b**) 5.5; and (**c**) 8.5 GHz.

Figure 10a–c highlights the simulated surface current distribution patterns of the proposed quad-port antenna at rejection frequencies 3.5, 5.5, and 8.5 GHz, respectively, on excitation of all four ports simultaneously. It is observed in Figure 10a that the current is mostly concentrated along the L-shaped slit, which is accountable for rejecting the Wi-MAX band. In the same way, the current is strong close to the outer split-ring (Figure 10b) and inner split-ring (Figure 10c), which are responsible for the WLAN and X-band rejection behavior, respectively.

The ECC between port-1 and port-2 of a four-port MIMO system can be computed using the expression [26]:

(7) $$\rho_{e}=\frac{{\left|S_{11}^{*}S_{12}+\;S_{21}^{*}S_{22}+S_{13}^{*}S_{32}+\;S_{14}^{*}S_{42}\right|}^{2}}{\left(1-{\left|S_{11}\right|}^{2}-{\left|S_{21}\right|}^{2}-{\left|S_{31}\right|}^{2}-{\left|S_{41}\right|}^{2}\right)\left(1-{\left|S_{12}\right|}^{2}-{\left|S_{22}\right|}^{2}-{\left|S_{32}\right|}^{2}-{\left|S_{42}\right|}^{2}\right)}$$

**Figure 11:**
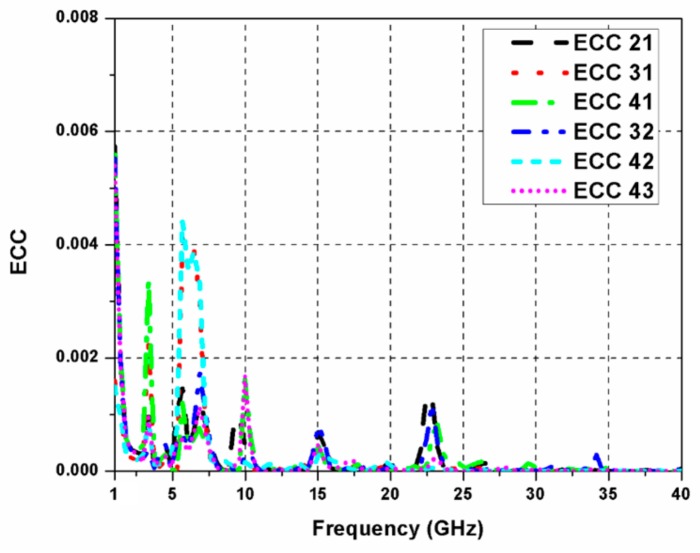
Envelope correlation coefficient (ECC) of the proposed quad-port SWB MIMO antenna.

**Figure 12:**
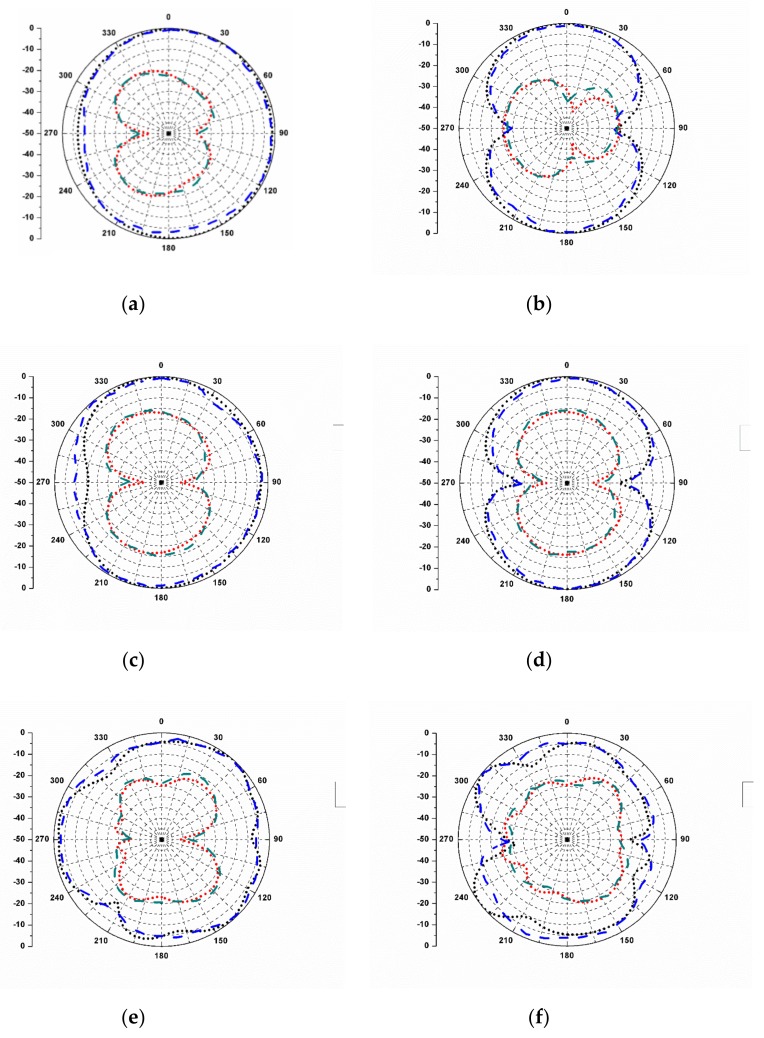
Radiation patterns of the proposed antenna: (**a**) H-plane/2.5 GHz; (**b**) E-plane/2.5 GHz; (**c**) H-plane/7.5 GHz; (**d**) E-plane/7.5 GHz; (**e**) H-plane/12 GHz; and (**f**) E-plane/12 GHz.

Similarly, ECC between other ports of the antenna can also be calculated. Figure 11 illustrates the ECC values between different antenna ports. It is noted that ECC remains below 0.01 for the complete SWB region. Figure 12 shows the simulated and measured co-polar and cross-polar radiation patterns of the proposed antenna at frequencies 2.5, 7.5, and 12 GHz. The difference between the levels of co-polar and cross-polar radiation patterns is greater than 15 dB in both the E-plane and H-plane, which signifies stability in the radiation performance of the antenna. It can also be noticed from the figure that the H-plane co-polar patterns show omnidirectional characteristics and E-plane co-polar patterns show bi-directional characteristics.

Table 2 gives a comparison of various parameters of the designed antenna and other similar antennas. The comparison shows that the proposed antenna configuration has several advantages over previously reported antennas [8,9,10,11,12,13,14,15,16,17,18,19,20,21], in terms of bandwidth ratio, compact size, number of radiating patches, and isolation among radiating elements. Further, the usage of CPW feeding in the proposed antenna provides the advantage of easy integration into portable devices. In the proposed antenna, the signals at the rejection frequencies (3.5, 5.5, and 8.5 GHz) are eliminated using CSRR and an L-shaped slit, without using any filtering circuitry/active devices. The use of filtering circuitry results in bulky design, and in turn, creates problems during the integration stage, due to the greater space requirement. Moreover, the radiating patches are arranged orthogonally and anti-parallel to provide polarization diversity and better isolation between antenna ports. A common ground plane is used in the proposed antenna to provide stable operation of the quad-port SWB MIMO antenna.

## 4. Conclusions

In this paper, a compact, tapered CPW-fed quad-port MIMO antenna with triple band-notched features was designed and developed. Self-complementarity was used to achieve SWB characteristics, and notch bands were attained by loading an L-shaped slit and CSRR in the antenna resonating element. The coplanar design of the radiators with connected ground planes offers a compact antenna structure that can be easily integrated into the portable device or monolithic microwave integrated circuits. The simulated and measured gain, isolation, S-parameters, and radiation patterns were investigated and verified. The performance of the proposed antenna for various communication bands like L, S, C, X, Ku, K, and Ka proves that it could be a good choice for wireless access systems, cognitive radio, radio astronomy, wideband high-definition television, and other short-range and long-range wireless, satellite and defense applications.

## Figures and Tables

**Table 1:** Proposed TSESC antenna dimensions.

Parameter	Dimension (mm)	Parameter	Dimension (mm)
*L*_1_	32.5	*R*_2_	7
*W*_1_	30.5	*s*_1_	12.4
*L*_2_	15.7	*s*_2_	1.9
*L*_3_	3.5	*s*_3_	0.4
*L*_4_	13.3	*u*_1_	0.5
*L*_5_	12.9	*u*_2_	0.5
*W*_2_	11	*c*_1_	3
*W*_3_	10	*c*_2_	2
*W*_4_	9.3	*g*_1_	1.3
*W*_5_	4.5	*g*_2_	2
*W*_6_	4.5	*g*_3_	2
*W*_7_	11.7	*L*_6_	63
*W*_8_	2.9	*L*_7_	63
*R*_1_	14.4	*L*_8_	19.5

**Table 2:** Comparison of different parameters of the designed antenna with existing designs.

Ref.	Number of Ports	Impedance Bandwidth (GHz)	Bandwidth Ratio	Antenna Size (mm^3^)	Number of Notch Bands	Notched Band Central Frequency (GHz)	Isolation (dB)	ECC
[8]	1	3.4–37.4	11:1	30 × 28 × 1.6	---	---	---	---
[9]	1	1–30	30:1	20 × 20 × 1	---	---	---	---
[10]	1	3.8–68	18:1	18.5 × 20 × 1.6	---	---	---	---
[11]	1	10–50	5:1	60 × 60 × 1.524	---	---	---	---
[12]	1	1.44–18.8	13:1	35 × 77 × 1.6	---	---	---	---
[13]	1	1.3–12	9:1	40 × 51.5 × 1.6	---	---	---	---
[14]	1	3–12	4:1	19 × 16 × 1.6	---	---	---	---
[15]	1	2.82–13.86	5:1	28.5 × 26 × 1.6	1	5.5	---	---
[16]	2	3–12	4:1	21 × 38 × 1.6	---	---	>15	<0.15
[17]	2	2.19–11.07	5:1	41 × 30 × 1	---	---	>20	<0.1
[18]	2	2.6–13	5:1	66.8 × 40 × 0.8	2	3.5, 5.5	>15	<0.02
[19]	2	2.2–11	5:1	30 × 41 × 1.59	2	5.5, 8.1	>20	<0.1
[20]	2	1.5–40	27:1	55.6 × 50.5 × 1.6	1	5.9–7.1	>20	<0.005
[21]	4	1.25–40	32:1	52 × 52 × 1.6	2	3.5, 5.5	>18	<0.09
Prop.	4	1.3–40	31:1	63 × 63 × 1.6	3	3.5, 5.5, 8.5	>16	<0.01
